# The Potential Use of Arsenic Trioxide in the Treatment of Systemic Lupus Erythematosus

**DOI:** 10.3390/ijms25179577

**Published:** 2024-09-04

**Authors:** Tsz Ching Mok, Chi Chiu Mok

**Affiliations:** 1 Department of Medicine, Ruttonjee Hospital, Hong Kong SAR, China; 2 Department of Medicine and Geriatrics, Tuen Mun Hospital, Hong Kong SAR, China

**Keywords:** arsenic trioxide, non-leukemic, autoimmune, immune-mediated, rheumatic, lupus

## Abstract

Arsenic trioxide (ATO) is now part of the standard regimen for the treatment of newly diagnosed and relapsed acute promyelocytic leukemia. The availability of an oral form of ATO has greatly reduced the incidence of cardiotoxicity as compared to intravenous (IV) administration. Increasing evidence suggests that ATO has anti-inflammatory properties that may be useful for the treatment of autoimmune diseases. These include the modulation of Treg cell activation, Th1/Th2 and Th17/Treg balance, depletion of activated T cells and plasmacytoid dendritic cells, and influence of B-cell differentiation, leading to reduced autoantibody and cytokine production. ATO has also been shown to induce apoptosis of activated fibroblast-like synoviocytes through the generation of reactive oxygen species and alter the gut microbiota in collagen-induced arthritis. Despite the emergence of newer treatment modalities, the treatment of systemic lupus erythematosus (SLE), especially refractory manifestations, remains a challenge, owing to the paucity of effective biological and targeted therapies that are devoid of adverse effects. Oral ATO is an attractive option for the treatment of SLE because of the lower cost of production, convenience of administration, and reduced cardiotoxicity. This article summarizes the anti-inflammatory mechanisms of ATO and its potential application in the treatment of SLE and other rheumatic diseases.

## 1. Introduction

Arsenic is an element commonly found in nature and exists in organic and inorganic forms. While arsenic and its compounds are well known to be a means of poisoning, their medicinal use dates back to ancient Greece and Rome when arsenic was viewed as both a therapeutic agent and a poison [1]. The arsenicals were probably introduced into Western medicine at around the eighteenth century [2] for the treatment of chronic leukemia. The utility of arsenic in the treatment of hematological malignancies is limited by its toxicities, in particular cardiological. Interest in the therapeutic use of intravenous (IV) arsenic trioxide (ATO) has recently been rekindled due to promising reports from mainland China highlighting its efficacy and cost effectiveness in acute promyelocytic leukemia (APL) [3].

The introduction of a combination regimen of all-trans retinoic acid (ATRA) and ATO in the 1990s has turned APL, a highly fatal condition, to a highly curable condition. ATO as a single agent can induce remission of ≥70% of APL patients whereas ATO combined with ATRA can induce remission of at least 90% of APL patients [4]. Trisenox, an injectable form of ATO, used in conjunction with ATRA, was approved by the United States (US) Food and Drug administration (FDA) in 2018 for the treatment of adults with newly diagnosed low-risk APL characterized by the presence of the t(15;17) translocation or promyelocytic leukemia/retinoic acid receptor alpha (PML/RARα) gene expression [5]. A phase III clinical trial from mainland China demonstrated that the overall survival rate for APL patients who received a combination of Trisenox and ATRA reached 96.6% at 3 years, with disease relapse occurring in less than 1% of the patients [6].

Although ATO is now part of the standard regimen for the treatment of newly diagnosed and relapsed APL [7,8], concerns about the IV preparation of ATO include inconvenience, need for hospitalization, vascular access, cost, as well as dose-related cardiotoxicity such as ventricular arrythmias and sudden cardiac death [9]. An oral solution of ATO is currently available in Hong Kong, China, with a bioavailability similar to that of the IV preparation [10,11]. The cardiac safety of the oral preparation is much more favorable than the IV preparation. Adverse effects of oral ATO in the treatment of acute leukemia are usually minor and respond to symptomatic treatment or temporary drug cessation [12]. These include skin reactions, gastrointestinal upset, and hepatitis. In a long-term observational study of oral ATO (10 mg/day) for the treatment of relapsed APL, the most commonly reported non-hematological AEs were elevation of liver parenchymal enzymes, headache, infections, skin rash, and gastrointestinal upset (nausea, vomiting) [13]. Three patients developed asymptomatic QTc prolongation. A more detailed study on cardiac rhythm in APL patients treated with oral ATO (10 mg/day) showed that transient prolongation of QTc to more than 500 ms occurred in 18% of patients 4 h after ingestion [14]. Premature ventricular beats were no more common in ATO users and none of the patients developed ventricular tachyarrhythmia.

## 2. Pharmacokinetics of Arsenic Trioxide

After IV administration, ATO is distributed throughout the body before it is metabolized in the liver. The drug undergoes hepatic methylation to become water-soluble methylated (MAs) and dimethylated (DMAs) metabolites, which are generally less toxic and excreted predominantly by the kidneys [15]. In a Japanese study [16], the plasma concentrations of inorganic arsenics (iAs) reached Cmax rapidly after IV administration of ATO on the first day. During repeated administration, the plasma concentrations of iAs reached a steady state but the mean total arsenic excretion rate (including iAs and methylated arsenic) was about 20% of the daily dose on the first day and remained at about 60% of the daily dose during the first to fourth week. Recent studies also demonstrated that several factors might affect the effective plasma concentration of ATO after IV administration [17,18,19]. These included polymorphisms of the gene encoding AS3MT that is important for methylation of the iAs [17,18], smoking, and prior chemotherapy exposure [19].

Arsenol is an oral ATO preparation available in Hong Kong and is primarily adopted for the treatment of APL. Compared to the IV preparation, oral ATO is absorbed into the bloodstream via the gastrointestinal tract, resulting in potentially variable bioavailability attributable to the first-pass metabolism in the liver. However, a previous study has revealed oral ATO can achieve systemic bioavailability comparable to that of IV ATO [11]. Similar to IV administration, orally absorbed ATO is methylated in the liver.

In addition to Arsenol, Realgar-Indigo naturalis formula (RIF) is another oral ATO formulation approved in mainland China for the treatment of APL [20]. RIF is a combination of four natural products: realgar, indigo naturalis, radix salviae miltiorrhizae, and radix pseudostellariae [21]. Realgar contains approximately 90% of tetra-arsenic tetra-sulfide (As4S4), an oral form of arsenic compound that was originally used as traditional Chinese medicine. RIF has demonstrated efficacy comparable to IV ATO in APL [6,22].

## 3. Mechanisms of Action of Arsenic Trioxide in Leukemia

APL is caused by a reciprocal chromosome translocation, t(15;17), that leads to the fusion of the PML gene on chromosome 15 with the RARα gene on chromosome 17. PML is a tumor-suppressor protein that binds to nuclear bodies (NBs), the key regulators of senescence, via its cysteine residues and enhances interaction with caspases to activate apoptosis. The formation of the PML/RARα oncoprotein impedes the differentiation of hematopoietic progenitor cells by disrupting transcriptional control and interfering with PML NBs that results in the blunting of p53 signaling and an increase in self-renewal of myeloid progenitors [23]. Moreover, the binding of PML/RARα to the retinoic acid response element (RARE) leads to recruitment of co-repressors and methylating enzymes, giving rise to epigenetic silencing, inhibition of granulocyte differentiation, and accumulation of abnormal promyelocytes [24].

**Figure 1:**
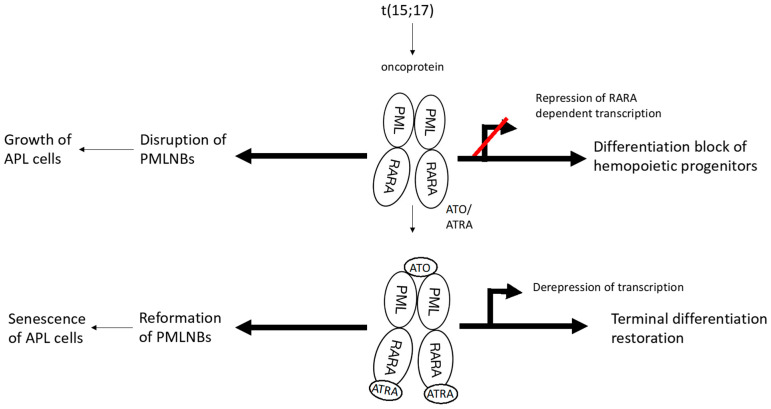
Mechanism of action of arsenic trioxide in promyelocytic leukemia. 1. Translocation t(15;17) leads to formation of the PML/RARα fusion oncoprotein; 2. This protein impedes the differentiation of myeloid progenitors by interfering with PML NBs; 3. ATO binds to PML-RARα and enhances SUMOylation and proteasomal degradation of PML/RARα; 4. Degradation of PML-RARα restores biogenesis of PML NBs, induces senescence of the APL cells, and restores terminal differentiation of the myeloid progenitors. Red line refers to “blocks” transcription and differentiation.

ATO induces partial differentiation and apoptosis in the APL cells through various molecular mechanisms [25]. The drug has a strong affinity for PML-RARα and after binding through the spatially proximate cysteine residues, it interacts with the small ubiquitin-like protein modifier (SUMO)-conjugating enzyme UBC9, resulting in enhancement of SUMOylation and proteasomal degradation of the fusion protein [26]. Degradation of PML-RARα restores biogenesis of PML NBs and induces apoptosis of the APL cells [27] (Figure 1).

Moreover, ATO interacts with mitochondrial proteins and disrupts the mitochondrial membrane potential, leading to the generation of reactive oxygen species (ROS), release of cytochrome c into the cytoplasm, and stimulation of apoptosis [28]. On the other hand, there is evidence to show that ATO’s effects on cellular apoptosis and partial differentiation depends on its concentrations. At low concentrations (0.1–0.5 µmol/L), ATO fosters differentiation of APL cells. However, at higher concentrations (0.5–2 µmol/L), it activates the intrinsic apoptotic cell death pathway [29].

In addition, ATO has been shown to exhibit in vitro anti-cancer and anti-angiogenic activities in cell lines of liver, pancreatic, and prostatic cancers [30,31,32]. It is hypothesized that ATO reduces the production of angiogenic cytokines and induces apoptosis of tissue and endothelial cells [33].

## 4. Anti-Inflammatory Effects of Arsenic Trioxide

The multiple effects of ATO on cellular apoptosis, autophagy, differentiation, growth, angiogenesis, cytokine production, and functions of the immune cells have extended its potential clinical use in other conditions beyond hematological malignancies. There is increasing evidence of the anti-inflammatory effects of ATO in murine models of systemic lupus erythematosus, systemic sclerosis, and rheumatoid arthritis [34,35,36,37,38,39,40,41,42,43,44,45]. These are summarized in Table 1.

### 4.1. Systemic Lupus Erythematosus

The MRL/lpr lupus mice spontaneously develop a lupus-like syndrome that includes skin lesions, glomerulonephritis, and vasculitis due to inactivation of Fas-mediated apoptosis. Peritoneal injection of these mice with ATO caused a rapid and significant reduction in the size of the spleen and lymph nodes, and suppressed skin lesions as compared to the control mice [34]. Autoantibody production, cytokines, and immune complex deposition in the kidney was also reduced. Furthermore, ATO administration delayed the onset of nephritis and significantly improved the survival of these mice through activation of caspases and elimination of activated T cells responsible for lymphoproliferation and the lesions in skin, lung and the kidneys [34].

The lupus prone BXSB mice develop symptoms mimicking human SLE that is associated with autoantibody production, lymphoid activation and organ hyperplasia. A study evaluating the effects of tetra-arsenic tetra-sulfide (As4S4), another arsenic preparation, on the BXSB mice was performed. Treatment of these mice with As4S4 for 8 weeks resulted in reduction of splenomegaly and alleviation of skin, liver and kidney lesions with mild side effects. Histological analysis showed decreased immune complex deposition, mesangial proliferation, and inflammatory cell infiltration in the kidney and liver tissues. Moreover, As4S4 treatment also led to the inhibition of monocytosis in the spleen and decreased serum interleukin-6 (IL-6) level [35].

In another in vitro experiment, the effect of different concentrations of ATO on IFN-γ expression in the splenocytes of MRL/lpr mice and peripheral blood mononuclear cells (PBMCs) of human SLE patients was investigated [36]. ATO treatment was effective in reducing the mRNA and protein expression levels of IFN-γ in the mice splenocytes and human SLE PBMCs, which was accompanied by a reduction in histone H4 and H3 acetylation in the IFN-γ promoter and decreased combination of RNA polymerase II to the IFN-γ promoter.

### 4.2. Systemic Sclerosis

In a murine model of hypochlorite-induced systemic sclerosis (SSc), daily intraperitoneal injections of ATO for 6 weeks limited dermal thickness and inhibited collage deposition as assessed by histological examination [37]. ATO treatment reduced vascular cell adhesion molecule 1 level and inhibited the autoantibody, IL-4 and IL-13 production by activated T cells. These beneficial effects of ATO were mediated through reactive oxygen species (ROS) generation that selectively killed activated fibroblasts. In an experimental model of chronic graft-versus-host disease (GVHD) induced by body radiation, followed by bone marrow and spleen cell transplantation, mice developed severe clinical symptoms that included diarrhea, alopecia, vasculitis, and fibrosis of the skin and internal organs [38]. Daily intraperitoneal injection of ATO to the mice abrogated these symptoms. The positive effects of ATO in this GVHD model were mediated by elimination of activated CD4+ T cells and plasmacytoid dendritic cells (pDCs) through depletion of glutathione [38].

The pDCs are a unique subset of dendritic cells that secrete high levels of type I IFNs that are important in the pathophysiology of SSc and SLE. High concentrations of ATO were shown to induce apoptosis of pDCs derived from untreated patients with SSc via the mitochondrial pathway [39]. At clinically relevant concentrations, ATO preferentially inhibited IFN-*α* secretion and phosphorylation of the interferon regulatory factor 7. In addition, the capacity of pDCs to induce CD4+ T-cell proliferation, Th1/Th2 polarization, and B-cell differentiation to plasmablasts was also downregulated by ATO administration.

In the hypochlorite-induced SSc murine model, copper ions combined with ATO significantly reduced skin thickening and cutaneous fibrosis in a manner equivalent to monotherapy with a double dose of ATO to induce the same immunological effects, which included a decrease in the number of B cells and reduced activation of CD4+ T cells [40]. Moreover, co-treatment of copper and ATO increased ROS production and enhanced apoptosis of murine fibroblasts compared to ATO alone.

The Fra2 transgenic mice (Fra2^TG^) mimics human SSc because they develop severe vascular remodeling of the pulmonary arterioles (pulmonary hypertension) and interstitial pneumonitis-like lung disease. A study showed that ATO treatment of these mice, as opposed to control mice, resulted in improvement of the lung histology and reduction of CD4+ T-cell infiltration, a trend of reduction in fibrotic markers and a strong reduction in vascular remodeling [41]. RNA-sequencing analysis of lung tissues from ATO-treated mice revealed a downregulation of biological pathways associated with activity of the immune pathways, such as T-cell activation, regulation of leucocyte activation, leucocyte cell–cell adhesion, and regulation of lymphocyte activation.

### 4.3. Inflammatory Arthritis

In addition, ATO has been tested in the murine models of rheumatoid arthritis (RA). Fibroblast-like synoviocytes (FLS) are important cells that perpetuate inflammation in the joints and lead to structural damage. In a murine model of collagen-induced arthritis (CIA), ATO significantly enhanced the apoptosis of the (FLS) and contributed to histological recovery in the synovial membrane, along with inhibition of synovial hyperplasia and inflammation in the joints [42]. ATO may also alleviate inflammatory arthritis through the modulation of the balance in the Th17/Treg and Th1/Th2 pathways [43,44]. In an experiment, peripheral blood and synovial mononuclear cells were isolated from treatment-naïve RA patients. In vitro administration of ATO resulted in the inhibition of Th17 differentiation through a reduction of STAT3 mRNA expression and enhancement of Treg cell generation through an augmentation of Foxp3 expression in these cells [33]. ATO was also shown to downregulate the Th1/Th2 ratio [33] and improve the Th17/Treg balance in the CIA mice [46]. The same group of investigators also demonstrated by single-cell RNA sequencing that ATO modulated several genes associated with inflammation, activation, and differentiation of the peripheral blood Treg cells of treatment-naïve RA patients [43,44].

More recently, there is evidence that ATO ameliorates CIA by altering the gut microbiota. Fecal samples from CIA and control mice were collected for 16S rDNA gene sequencing and metabolomic analysis [45]. Compared with control mice, CIA mice showed differences in the composition of gut microbiota in both the phylum and genus level, as well as in many metabolites, including benzoic acid and (s)-2-acetolactate. These alterations were partially reversed by ATO treatment in the CIA mice, indicating that modulation of the gut microbiota and improvement in fecal metabolite abnormalities may be one mechanism of ATO in inflammatory arthritis.

## 5. Prospect of Arsenic Trioxide Treatment for SLE

### 5.1. Unmet Needs in the Treatment of SLE

Despite the improved life expectancy of patients with SLE over a few decades, further improvement in survival is hindered by the lack of blockbuster therapies in SLE [47]. Meta-analyses have shown an increase in the survival rates of SLE and lupus nephritis from the 1950s to 1990s but these plateaued off after the mid-1990s [48,49]. In low/middle-income countries, the 5-year and 10-year survival is still suboptimal. Refractory manifestations and treatment-related complications, especially those related to glucocorticoids (GCs), are the major causes of organ damage, mortality, and impaired quality of life in SLE patients [50].

Two novel biological agents have recently been introduced for the treatment of SLE. They are belimumab, a monoclonal antibody against B-cell activation factor (BAFF) [51,52] and anifrolumab, a monoclonal antibody against the type I interferon receptors [53,54]. Belimumab has been studied in active non-renal SLE and shows a benefit when added to the standard of care (SOC) therapies across different ethnic groups [55]. The recent BLISS-LN RCT showed a significantly higher renal response rate at week 104 when IV belimumab was added to the SOC therapies (MMF or low-dose cyclophosphamide in conjunction with GCs) for active lupus nephritis [51]. Although the effect size of belimumab in achieving the non-renal or renal end points in the pivotal RCTs is not impressively high, long-term data beyond 8 years showed that continuous use of the biological agent reduced flares, organ damage, and was GC sparing [56,57].

Anifrolumab has been studied in non-renal SLE in two pivotal RCTs [53,54]. Improvement in SLE activity, as assessed by the British Isles Lupus Assessment Group (BILAG)-based Composite Lupus Assessment (BICLA) response, was significantly more common when IV anifrolumab was added to the SOC therapies. Organ-specific data, such as the SLE skin activity score, showed a greater improvement with treatment than placebo after 52 weeks [54]. Extended data at 4 years showed a sustained benefit of the drug in non-renal SLE without new safety signals [58]. Despite phase II data showing a potential benefit of anifrolumab in lupus nephritis [59,60], further phase III clinical studies are needed to confirm the findings.

Despite the above evidence, the early use of biological agents as upfront combination therapy in SLE and lupus nephritis remains controversial [61]. Moreover, the availability and reimbursement of these biological agents in less affluent countries remains problematic. As a result, there is an unmet need to develop new treatment strategies with an enhanced efficacy-to-toxicity ratio and cost effectiveness in patients with SLE.

### 5.2. Disease Modifying Effects of SLE Therapies

Although disease modification has long been the concept of therapies of rheumatic diseases, its definition in SLE has not been well established. An international taskforce has suggested a conceptual framework to define the disease-modifying effect of a treatment modality in SLE. These include the following: (1) improvement in disease activity of SLE with minimal treatment-associated toxicity; the absence of major flares; and a reduction in dosages of GC and immunosuppressive drugs during the first year of treatment; (2) continued improvement in disease activity of SLE, immunosuppression-sparing effect, and the absence of flares during year 2–5 after therapy; and (3) the absence of new/worsening organ damage, including eGFR decline by 30% or more, throughout treatment to beyond 5 years [62].

A recent review of 32 selected clinical trials and 54 observational studies showed that 8 out of 14 SLE medications across different therapeutic classes met at least one of the criteria for disease modification up to 5 years [63]. While hydroxychloroquine improved overall survival beyond 5 years, no data on specific organ systems were reported. Only hydroxychloroquine and belimumab met disease modification definitions across three time points and beyond 5 years.

### 5.3. Novel Small Molecules for SLE

Novel small molecules targeting specific intracellular mechanisms of immune cells are being developed for the treatment of SLE [64]. The Janus kinases (JAKs), Bruton’s tyrosine kinases (BTKs), and spleen tyrosine kinases (SYKs) are important enzymes for activating receptor-mediated downstream signals from cytokines, growth factors, hormones, and Fc/CD40/B-cell receptors [65]. Inhibition of these kinases impairs cellular activation and differentiation, leading to diminished actions of the inflammatory cytokines. Modulation of intracellular protein degradation in immunoproteasomes leads to depletion of long-lived plasma cells and reduced production of interferon (IFN)-α and autoantibodies. Interference of the sphingosine 1-phosphate (S1P)/S1P receptor-1 (S1PR1) pathway limits trafficking of autoreactive lymphocytes, enhances Treg functions, and leads to decreased production of autoantibodies and the type I IFNs.

Although these targeted molecules are orally active and have the advantages of administration convenience and higher acceptance, lower production cost, and the absence of immunogenicity, they are still in the experimental stages, and none has yet been approved for the treatment of SLE. Moreover, phase II trials of some of the JAK, BTK, and SYK inhibitors in SLE were either negative or prematurely terminated [64]. Recent large phase III trials of baricitinib in non-renal SLE also failed to show clinical benefits [66,67]. While we are waiting for the results of ongoing phase III trials of other small molecules with promising phase II data (eg. deucravacitinib, zetomipzomib and cenerimod) [64,68], exploration of alternative novel oral drugs for SLE is of paramount importance.

### 5.4. Preliminary Clinical Data of ATO in SLE

Clinical studies of ATO in rheumatic diseases are generally lacking despite the promising mechanisms of the drug in preclinical and murine studies. Recently, a pilot 24-week phase IIa trial from France has shown an acceptable safety profile of IV ATO (0.1–0.2 mg/kg for 10 doses) in 11 patients with refractory SLE [69]. Four serious AEs occurred (grade 3 neutropenia, osteitis, neuropathy), two of which were related to ATO (neutropenia in two patients treated with higher doses of ATO and concomitant mycophenolate mofetil), leading to discontinuation of ATO. Neutrophil count recovered in these two patients without infective complications. Eighteen mild to moderate AEs were reported, which included hypomagnesemia, hypokalemia, diarrhea, and asymptomatic moderate QT prolongation (<470 ms). Regarding efficacy, half of the patients achieved the SLE response index-4 (SRI-4) and the mean corticosteroid dosage was decreased from 11.3 mg/day at baseline to 6 mg/day at week 24. Six patients achieved an SLE low-disease activity state at week 52, which persisted at the last follow-up (median 3 years, range 2–4 years). Titers of anti-dsDNA showed a trend of reduction after ATO therapy but levels of C3/4 and immunoglobulins were not altered. Pharmacokinetic study did not show correlation between plasma ATO levels and safety or efficacy.

The availability of oral forms of ATO with a better safety profile than the IV preparation and their preliminary benefits in the murine models of SLE warrants further attention in human SLE disease. As there are still no published studies of oral ATO in SLE, further clinical studies in SLE are necessary to establish its safety and efficacy. SLE patients with non-organ threatening disease that is refractory to standard conventional therapies are ideal candidates for a new placebo-controlled randomized controlled trial.

### 5.5. Clinical Experience of ATO in Other Rheumatic Diseases

To our knowledge, ATO has not been clinically tested in other human rheumatic diseases other than SLE [69] as described above. In a report of 17 patients with APL and concomitant skin psoriasis, an ATO-based induction regimen resulted in improvement of skin lesions in more than 80% of patients over a median follow-up period of 27 months [70]. The efficacy of oral ATO on skin psoriasis and psoriatic arthritis has to be further explored.

## 6. Conclusions and Outlook

The development of novel therapies in SLE has lagged behind other inflammatory rheumatic diseases. While there are a myriad of biologic and targeted disease-modifying anti-rheumatic drugs (DMARDs) that are approved for rheumatoid arthritis, spondyloarthritis, and psoriatic arthritis, only two biological agents have hitherto been approved for the treatment of SLE and lupus nephritis. Many biologics and small molecules in SLE have halted further development due to the failure of clinical trials to meet their primary efficacy end points [64]. However, the improvement in study design, patient stratification, and adjustment of background immunosuppression, as well as modification of disease activity assessment tools has facilitated future clinical trials in SLE. Although the Tyk-2 inhibitor, deucravacitinib, showed promise in a recent phase II trial [71], the concern of thromboembolism and cancer risk in post-marketing studies of RA, particularly in older patients with a cardiovascular risk [72], has led to caution of the use of Jakinibs in patients with SLE, who are at augmented risk of malignancy and premature atherosclerosis. Thus, there is an urgent and continued need to develop new therapies for SLE, particularly oral agents that are more acceptable to patients when compared to IV or subcutaneous drugs.

Arsenic and its compounds are historically known to be both a poison and a medicine. IV ATO has been used for the treatment of leukemia since the eighteenth century, but further development is hindered by its cardiac toxicities. With the introduction of an oral form of ATO, which is associated with a more favorable toxicity profile, it has become part of the standard therapies for APL. The anti-inflammatory properties of ATO in various murine models of rheumatic diseases also suggest its potential use in the treatment of human autoimmune diseases. In particular, the ex vivo and in vitro effects of ATO on modulation of Treg cell activation, depletion of activated T cells and dendritic cells, regulation of Th1/Th2 and Th17/Treg balance, reduction in autoantibody and cytokine production, and alteration of the gut microbiota, as well as the benefits observed from murine studies indicate that ATO worth further exploration in human SLE. Preliminary experience in refractory SLE revealed efficacy of IV ATO with an acceptable safety profile. As the oral preparations of ATO are associated with even fewer toxicities, further clinical trials of this drug in non-organ threatening SLE and other rheumatic diseases are eagerly awaited.

## Figures and Tables

**Table 1:** Immune mechanisms of arsenic trioxide in autoimmune rheumatic conditions.

Authors, Years	Model	Clinical Effects	Immunological Effects
Bobé et al., 2006 [34]	Lupus mice	↓ lymphoproliferation, skin, lung and kidney inflammation; significantly prolonged survival	Induced apoptosis and depletion of auto-reactive T cells, ↓ production of autoantibodies and cytokines
Zhao et al., 2013 [35]	Lupus mice	↓ splenomegaly, amelioration of skin, liver and renal lesions	↓ immune complex deposition, mesangial proliferation, and inflammatory cell infiltration in kidney and liver tissues, ↓monocytosis in spleen and serum interleukin-6 level
Hu et al., 2018 [36]	Lupus mice and blood cells from SLE patients	In vitro experiments	↓ mRNA and protein expression of IFN-γ in mice splenocytes and human SLE PBMCs
Kavian et al., 2012 [37]	Hypochlorite induced SSc mice	↓ dermal thickness and collagen deposition in skin and lung tissues	↓ vascular cell adhesion molecule 1 level, autoantibody, IL-4 and IL-13 production by activated T cells; selectively killed activated fibroblasts through ROS generation
Kavian et al., 2012 [38]	Induced chronic GVHD in mice	↓ GVHD symptoms, fibrosis of skin and internal organs	↓ activated CD4+ T cells and plasmacytoid dendritic cells (pDCs) through depletion of glutathione
Ye et al., 2020 [39]	Blood cells from SSc patients	In vitro experiments	Induced apoptosis of pDCs, preferentially inhibited IFN-α secretion and phosphorylation of the interferon regulatory factor 7, ↓ capacity of pDCs to induce CD4+ T-cell proliferation, Th1/Th22 polarization and B-cell differentiation to plasmablasts
Cauvet et al., 2023 [40]	Pre-clinical SSc mice	Improvement in lung histology, trend of reduction in fibrosis markers and strong reduction in vascular remodeling	↓ memory T cells, ↑ % of naive T cells in the lungs; downregulated biological pathways associated with activity of the immune pathways, such as T-cell activation, regulation of leucocyte activation, leucocyte cell–cell adhesion, and regulation of lymphocyte activation.
Chêne et al., 2023 [41]	Hypochlorite induced SSc mice	↓ skin thickening and fibrosis	↓ number of B cells and activation of CD4+ T cells, ↑ ROS production and apoptosis of murine fibroblasts
Mei et al., 2011 [42]	Collagen-induced arthritis in mice	↓ synovial hyperplasia and inflammation in the joints	Enhanced apoptosis of fibroblast-like synoviocytes
Li et al., 2019 [43]	Blood and synovial cells from RA patients	In vitro experiments	↓ Th17 differentiation through a reduction of STAT3 mRNA expression, ↑ Treg cell generation through an augmentation of Foxp3 expression, downregulated the Th1/Th2 ratio
Li et al., 2021 [44]	Treg cells from early RA patients	In vitro experiments	Modulated expression of several genes associated with inflammation, Treg-cell activation, and differentiation
Niu et al., 2022 [45]	Collagen-induced arthritis in mice	↓ arthritis	Modulated gut microbiota and improved fecal metabolite abnormalities

↑ means “increase”; ↓ means “decrease”.

## Data Availability

Not applicable as this is a review article. There are no raw data involved.

## Abbreviations

PML	Promyelocytic leukemia
RARα	Retinoic acid receptor alpha
NB	Nuclear bodies
ATO	Arsenic trioxide
SUMO	Small ubiquitin-like protein modifier
ATRA	All-trans retinoic acid

## References

[1] Miller W.H., Schipper H.M., Lee J.S., Singer J., Waxman S. (2002). Mechanisms of action of arsenic trioxide. Cancer Res..

[2] Kumana C.R., Mak R., Kwong Y.-L., Gill H. (2020). Resurrection of Oral Arsenic Trioxide for Treating Acute Promyelocytic Leukaemia: A Historical Account from Bedside to Bench to Bedside. Front. Oncol..

[3] Chen X., Hong Y., Zheng P., You X., Feng J., Huang Z., Wang Y. (2020). The economic research of arsenic trioxide for the treatment of newly diagnosed acute promyelocytic leukemia in China. Cancer.

[4] Shen Z.X., Shi Z.Z., Fang J., Gu B.W., Li J.M., Zhu Y.M., Shi J.Y., Zheng P.Z., Yan H., Liu Y.F. (2004). All-trans retinoic acid/As_2_O_3_ combination yields a high quality remission and survival in newly diagnosed acute promyelocytic leukemia. Proc. Natl. Acad. Sci. USA.

[5] Drugs@FDA: FDA-Approved Drugs [Internet]. https://www.accessdata.fda.gov/scripts/cder/daf/index.cfm?event=overview.process&ApplNo=021248.

[6] Zhu H.-H., Wu D.-P., Jin J., Li J.-Y., Ma J., Wang J.-X., Jiang H., Chen S.-J., Huang X.-J. (2013). Oral Tetra-Arsenic Tetra-Sulfide Formula Versus Intravenous Arsenic Trioxide as First-Line Treatment of Acute Promyelocytic Leukemia: A Multicenter Randomized Controlled Trial. J. Clin. Oncol..

[7] Sanz M.A., Fenaux P., Tallman M.S., Estey E.H., Löwenberg B., Naoe T., Lengfelder E., Döhner H., Burnett A.K., Chen S.-J. (2019). Management of acute promyelocytic leukemia: Updated recommendations from an expert panel of the European LeukemiaNet. Blood.

[8] Kantarjian H.M., Jain N., Garcia-Manero G., Welch M.A., Ravandi F., Wierda W.G., Jabbour E.J. (2021). The cure of leukemia through the optimist’s prism. Cancer.

[9] Vineetha V.P., Raghu K.G. (2019). An Overview on Arsenic Trioxide-Induced Cardiotoxicity. Cardiovasc. Toxicol..

[10] Ravandi F., Koumenis I., Johri A., Tallman M., Roboz G.J., Strickland S., Garcia-Manero G., Borthakur G., Naqvi K., Meyer M. (2020). Oral arsenic trioxide ORH-2014 pharmacokinetic and safety profile in patients with advanced hematologic disorders. Haematologica.

[11] Kumana C., Au W., Lee N., Kou M., Mak R., Lam C., Kwong Y. (2002). Systemic availability of arsenic from oral arsenic-trioxide used to treat patients with hematological malignancies. Eur. J. Clin. Pharmacol..

[12] Au W.-Y., Kwong Y.-L. (2008). Arsenic trioxide: Safety issues and their management. Acta Pharmacol. Sin..

[13] Gill H., Yim R., Lee H.K.K., Mak V., Lin S., Kho B., Yip S., Lau J.S.M., Li W., Ip H. (2018). Long-term outcome of relapsed acute promyelocytic leukemia treated with oral arsenic trioxide-based reinduction and maintenance regimens: A 15-year prospective study. Cancer.

[14] Siu C.-W., Au W.-Y., Yung C., Kumana C.R., Lau C.-P., Kwong Y.-L., Tse H.-F. (2006). Effects of oral arsenic trioxide therapy on QT intervals in patients with acute promyelocytic leukemia: Implications for long-term cardiac safety. Blood.

[15] Jansen R.J., Argos M., Tong L., Li J., Rakibuz-Zaman M., Islam T., Slavkovich V., Ahmed A., Navas-Acien A., Parvez F. (2016). Determinants and Consequences of Arsenic Metabolism Efficiency among 4,794 Individuals: Demographics, Lifestyle, Genetics, and Toxicity. Cancer Epidemiol. Biomark. Prev..

[16] Fujisawa S., Ohno R., Shigeno K., Sahara N., Nakamura S., Naito K., Kobayashi M., Shinjo K., Takeshita A., Suzuki Y. (2007). Pharmacokinetics of arsenic species in Japanese patients with relapsed or refractory acute promyelocytic leukemia treated with arsenic trioxide. Cancer Chemother. Pharmacol..

[17] Li J., Packianathan C., Rossman T.G., Rosen B.P. (2017). Nonsynonymous Polymorphisms in the Human AS3MT Arsenic Methylation Gene: Implications for Arsenic Toxicity. Chem. Res. Toxicol..

[18] Lu J., Hu S., Wang W., Li J., Dong Z., Zhou J., Hai X. (2018). AS3MT Polymorphisms, Arsenic Metabolism, and the Hematological and Biochemical Values in APL Patients Treated with Arsenic Trioxide. Toxicol. Sci..

[19] Ghiuzeli C.M., Stýblo M., Saunders J., Calabro A., Budman D., Allen S., Devoe C., Dhingra R. (2022). The pharmacokinetics of therapeutic arsenic trioxide in acute promyelocytic leukemia patients. Leuk. Lymphoma.

[20] Lou Y., Ma Y., Jin J., Zhu H. (2021). Oral Realgar-Indigo Naturalis Formula Plus Retinoic Acid for Acute Promyelocytic Leukemia. Front. Oncol..

[21] Zhu H.-H., Hu J., Lo-Coco F., Jin J. (2019). The simpler, the better: Oral arsenic for acute promyelocytic leukemia. Blood.

[22] Zhu H.-H., Wu D.-P., Du X., Zhang X., Liu L., Ma J., Shao Z.-H., Ren H.-Y., Hu J.-D., Xu K.-L. (2018). Oral arsenic plus retinoic acid versus intravenous arsenic plus retinoic acid for non-high-risk acute promyelocytic leukaemia: A non-inferiority, randomised phase 3 trial. Lancet Oncol..

[23] de Thé H., Pandolfi P.P., Chen Z. (2017). Acute Promyelocytic Leukemia: A Paradigm for Oncoprotein-Targeted Cure. Cancer Cell..

[24] Chin L., Kumana C.R., Kwong Y.-L., Gill H. (2022). The Development and Clinical Applications of Oral Arsenic Trioxide for Acute Promyelocytic Leukaemia and Other Diseases. Pharmaceutics.

[25] Diaz Z., Colombo M., Mann K.K., Su H., Smith K.N., Bohle D.S., Schipper H.M., Miller W.H. (2005). Trolox selectively enhances arsenic-mediated oxidative stress and apoptosis in APL and other malignant cell lines. Blood.

[26] Zhang X.-W., Yan X.-J., Zhou Z.-R., Yang F.-F., Wu Z.-Y., Sun H.-B., Liang W.-X., Song A.-X., Lallemand-Breitenbach V., Jeanne M. (2010). Arsenic trioxide controls the fate of the PML-RARalpha oncoprotein by directly binding PML. Science.

[27] Yan M., Wang H., Wei R., Li W. (2024). Arsenic trioxide: Applications, mechanisms of action, toxicity and rescue strategies to date. Arch. Pharm. Res..

[28] Liu F., Deng Y., Wang A., Yang T., Ke H., Tang Y., Wu H., Chen H. (2024). Harness arsenic in medicine: Current status of arsenicals and recent advances in drug delivery. Expert Opin. Drug Deliv..

[29] Chen G.Q., Shi X.G., Tang W., Xiong S.M., Zhu J., Cai X., Han Z.G., Ni J.H., Shi G.Y., Jia P.M. (1997). Use of arsenic trioxide (As_2_O_3_) in the treatment of acute promyelocytic leukemia (APL): I. As_2_O_3_ exerts dose-dependent dual effects on APL cells. Blood.

[30] Ji H., Li Y., Jiang F., Wang X., Zhang J., Shen J., Yang X. (2014). Inhibition of transforming growth factor beta/SMAD signal by MiR-155 is involved in arsenic trioxide-induced anti-angiogenesis in prostate cancer. Cancer Sci..

[31] Gao J.-K., Wang L.-X., Long B., Ye X.-T., Su J.-N., Yin X.-Y., Zhou X.-X., Wang Z.-W. (2015). Arsenic Trioxide Inhibits Cell Growth and Invasion via Down-Regulation of Skp2 in Pancreatic Cancer Cells. Asian Pac. J. Cancer Prev. APJCP.

[32] Jiang F., Wang X., Liu Q., Shen J., Li Z., Li Y., Zhang J. (2014). Inhibition of TGF-β/SMAD3/NF-κB signaling by microRNA-491 is involved in arsenic trioxide-induced anti-angiogenesis in hepatocellular carcinoma cells. Toxicol. Lett..

[33] Zhang J., Zhang Y., Wang W., Zhang Z. (2019). Potential molecular mechanisms underlying the effect of arsenic on angiogenesis. Arch. Pharmacal Res..

[34] Bobé P., Bonardelle D., Benihoud K., Opolon P., Chelbi-Alix M.K. (2006). Arsenic trioxide: A promising novel therapeutic agent for lymphoproliferative and autoimmune syndromes in MRL/lpr mice. Blood.

[35] Zhao Y., Wen G., Qiao Z., Xu H., Sun Q., Huang H., Shan S., Mu Z., Zhang J. (2013). Effects of tetra-arsenic tetra-sulfide on BXSB lupus-prone mice: A pilot study. Lupus.

[36] Hu H., Chen E., Li Y., Zhu X., Zhang T., Zhu X. (2018). Effects of Arsenic Trioxide on INF-gamma Gene Expression in MRL/lpr Mice and Human Lupus. Biol. Trace Elem. Res..

[37] Kavian N., Marut W., Servettaz A., Nicco C., Chéreau C., Lemaréchal H., Borderie D., Dupin N., Weill B., Batteux F. (2012). Reactive oxygen species–mediated killing of activated fibroblasts by arsenic trioxide ameliorates fibrosis in a murine model of systemic sclerosis. Arthritis Rheum..

[38] Kavian N., Marut W., Servettaz A., Laude H., Nicco C., Chéreau C., Weill B., Batteux F. (2012). Arsenic Trioxide Prevents Murine Sclerodermatous Graft-versus-Host Disease. J. Immunol..

[39] Ye Y., Ricard L., Siblany L., Stocker N., De Vassoigne F., Brissot E., Lamarthée B., Mekinian A., Mohty M., Gaugler B. (2020). Arsenic trioxide induces regulatory functions of plasmacytoid dendritic cells through interferon-α inhibition. Acta Pharm. Sin. B.

[40] Cauvet A., Decellas A., Guignabert C., Rongvaux-Gaïda D., Thuillet R., Ottaviani M., Tu L., Rieger F., Avouac J., Allanore Y. (2023). Arsenic trioxide demonstrates efficacy in a mouse model of preclinical systemic sclerosis. Arthritis Res. Ther..

[41] Chêne C., Rongvaux-Gaïda D., Thomas M., Rieger F., Nicco C., Batteux F. (2023). Optimal combination of arsenic trioxide and copper ions to prevent autoimmunity in a murine HOCl-induced model of systemic sclerosis. Front. Immunol..

[42] Mei Y., Zheng Y., Wang H., Gao J., Liu D., Zhao Y., Zhang Z. (2011). Arsenic Trioxide Induces Apoptosis of Fibroblast-like Synoviocytes and Represents Antiarthritis Effect in Experimental Model of Rheumatoid Arthritis. J. Rheumatol..

[43] Li C., Zhang J., Wang W., Wang H., Zhang Y., Zhang Z. (2019). Data on arsenic trioxide modulates Treg/Th17/Th1/Th2 cells in treatment-naïve rheumatoid arthritis patients and collagen-induced arthritis model mice. Data Brief.

[44] Li C., Chu T., Zhang Z., Zhang Y. (2021). Single Cell RNA-Seq Analysis Identifies Differentially Expressed Genes of Treg Cell in Early Treatment-Naive Rheumatoid Arthritis by Arsenic Trioxide. Front. Pharmacol..

[45] Niu S., Zhu X., Zhang J., Ma Y., Lang X., Luo L., Li W., Zhao Y., Zhang Z. (2022). Arsenic trioxide modulates the composition and metabolic function of the gut microbiota in a mouse model of rheumatoid arthritis. Int. Immunopharmacol..

[46] Li C., Zhang J., Wang W., Wang H., Zhang Y., Zhang Z. (2019). Arsenic trioxide improves Treg and Th17 balance by modulating STAT3 in treatment-naïve rheumatoid arthritis patients. Int. Immunopharmacol..

[47] Mok C.C., Ho L.Y., Chan K.L., Tse S.M., To C.H. (2020). Trend of Survival of a Cohort of Chinese Patients with Systemic Lupus Erythematosus over 25 Years. Front. Med..

[48] Tektonidou M.G., Lewandowski L.B., Hu J., Dasgupta A., Ward M.M. (2017). Survival in adults and children with systemic lupus erythematosus: A systematic review and Bayesian meta-analysis of studies from 1950 to 2016. Ann. Rheum. Dis..

[49] Tektonidou M.G., Dasgupta A., Ward M.M. (2016). Risk of End-Stage Renal Disease in Patients with Lupus Nephritis, 1971–2015: A Systematic Review and Bayesian Meta-Analysis. Arthritis Rheumatol..

[50] Mok C.C., Ho L.Y., Tse S.M., Chan K.L. (2017). Prevalence of remission and its effect on damage and quality of life in Chinese patients with systemic lupus erythematosus. Ann. Rheum. Dis..

[51] Furie R., Rovin B.H., Houssiau F., Malvar A., Teng Y.O., Contreras G., Amoura Z., Yu X., Mok C.-C., Santiago M.B. (2020). Two-Year, Randomized, Controlled Trial of Belimumab in Lupus Nephritis. N. Engl. J. Med..

[52] Navarra S.V., Guzmán R.M., Gallacher A.E., Hall S., Levy R.A., Jimenez R.E., Li E.K., Thomas M., Kim H.Y., León M.G. (2011). Efficacy and safety of belimumab in patients with active systemic lupus erythematosus: A randomised, placebo-controlled, phase 3 trial. Lancet.

[53] Furie R.A., Morand E.F., Bruce I.N., Manzi S., Kalunian K.C., Vital E.M., Ford T.L., Gupta R., Hiepe F., Santiago M. (2019). Type I interferon inhibitor anifrolumab in active systemic lupus erythematosus (TULIP-1): A randomised, controlled, phase 3 trial. Lancet Rheumatol..

[54] Morand E.F., Furie R., Tanaka Y., Bruce I.N., Askanase A.D., Richez C., Bae S.-C., Brohawn P.Z., Pineda L., Berglind A. (2020). Trial of Anifrolumab in Active Systemic Lupus Erythematosus. N. Engl. J. Med..

[55] Mok C.C. (2017). Therapeutic monitoring of the immuno-modulating drugs in systemic lupus erythematosus. Expert Rev. Clin. Immunol..

[56] van Vollenhoven R.F., Navarra S.V., Levy R.A., Thomas M., Heath A., Lustine T., Adamkovic A., Fettiplace J., Wang M.-L., Ji B. (2020). Long-term safety and limited organ damage in patients with systemic lupus erythematosus treated with belimumab: A Phase III study extension. Rheumatology.

[57] Urowitz M.B., Aranow C., Asukai Y., Bass D.L., Bruce I.N., Chauhan D., Dall’Era M., Furie R., Fox N.L., Gilbride J.A. (2022). Impact of Belimumab on Organ Damage in Systemic Lupus Erythematosus. Arthritis Care Res..

[58] Kalunian K.C., Furie R., Morand E.F., Bruce I.N., Manzi S., Tanaka Y., Winthrop K., Hupka I., Zhang L., Werther S. (2023). A Randomized, Placebo-Controlled Phase III Extension Trial of the Long-Term Safety and Tolerability of Anifrolumab in Active Systemic Lupus Erythematosus. Arthritis Rheumatol..

[59] Jayne D., Rovin B., Mysler E.F., Furie R.A., Houssiau F.A., Trasieva T., Knagenhjelm J., Schwetje E., Chia Y.L., Tummala R. (2022). Phase II randomised trial of type I interferon inhibitor anifrolumab in patients with active lupus nephritis. Ann. Rheum. Dis..

[60] Jayne D., Rovin B., Mysler E., Furie R., Houssiau F., Trasieva T., Knagenhjelm J., Schwetje E., Tang W., Tummala R. (2023). Anifrolumab in lupus nephritis: Results from second-year extension of a randomised phase II trial. Lupus Sci. Med..

[61] Mok C.C. (2023). Combination strategies for lupus nephritis: Facts and controversies. Expert Rev. Clin. Immunol..

[62] van Vollenhoven R., Askanase A.D., Bomback A.S., Bruce I.N., Carroll A., Dall’Era M., Daniels M., Levy R.A., Schwarting A., Quasny H.A. (2022). Conceptual framework for defining disease modification in systemic lupus erythematosus: A call for formal criteria. Lupus Sci. Med..

[63] Askanase A.D., Furie R.A., Dall’Era M., Bomback A.S., Schwarting A., Zhao M.-H., Bruce I.N., Khamashta M., Rubin B., Carroll A. (2024). Disease-modifying therapies in systemic lupus erythematosus for extrarenal manifestations. Lupus Sci. Med..

[64] Mok C.C. (2023). Targeted Small Molecules for Systemic Lupus Erythematosus: Drugs in the Pipeline. Drugs.

[65] Mok C.C. (2019). The Jakinibs in systemic lupus erythematosus: Progress and prospects. Expert Opin. Investig. Drugs.

[66] Morand E.F., Vital E.M., Petri M., van Vollenhoven R., Wallace D.J., Mosca M., Furie R.A., Silk M.E., Dickson C.L., Meszaros G. (2023). Baricitinib for systemic lupus erythematosus: A double-blind, randomised, placebo-controlled, phase 3 trial (SLE-BRAVE-I). Lancet.

[67] Petri M., Bruce I.N., Dörner T., Tanaka Y., Morand E.F., Kalunian K.C., Cardiel M.H., Silk M.E., Dickson C.L., Meszaros G. (2023). Baricitinib for systemic lupus erythematosus: A double-blind, randomised, placebo-controlled, phase 3 trial (SLE-BRAVE-II). Lancet.

[68] Mok C.C. (2024). Outlook of the jakinibs in systemic lupus erythematous after baricitinib failed. Int. J. Rheum. Dis..

[69] Hamidou M., Néel A., Poupon J., Amoura Z., Ebbo M., Sibilia J., Viallard J.-F., Gaborit B., Volteau C., Hardouin J.B. (2021). Safety and efficacy of low-dose intravenous arsenic trioxide in systemic lupus erythematosus: An open-label phase IIa trial (Lupsenic). Arthritis Res. Ther..

[70] Ge F., Zhang Y., Cao F., Li J., Hou J., Wang P., Li H., Xu M., Liu S., Li L. (2016). Arsenic trioxide-based therapy is suitable for patients with psoriasis-associated acute promyelocytic leukemia—A retrospective clinical study. Hematology.

[71] Morand E., Pike M., Merrill J.T., van Vollenhoven R., Werth V.P., Hobar C., Delev N., Shah V., Sharkey B., Wegman T. (2023). Deucravacitinib, a Tyrosine Kinase 2 Inhibitor, in Systemic Lupus Erythematosus: A Phase II, Randomized, Double-Blind, Placebo-Controlled Trial. Arthritis Rheumatol..

[72] Ytterberg S.R., Bhatt D.L., Mikuls T.R., Koch G.G., Fleischmann R., Rivas J.L., Germino R., Menon S., Sun Y., Wang C. (2022). Cardiovascular and Cancer Risk with Tofacitinib in Rheumatoid Arthritis. N. Engl. J. Med..

